# Interaction between Perivascular and Luminal Factors in Arteriovenous Fistula in Yucatan Miniswine

**Published:** 2025-03-11

**Authors:** Fanya Xia, Jacob Smith, Vikrant Rai, Devendra K Agrawal

**Affiliations:** Department of Translational Research, College of Osteopathic Medicine of the Pacific, Western University of Health Sciences, Pomona CA 91766, USA

**Keywords:** Apoptosis, Arteriovenous fistula, Immune cells, Inflammation, Luminal factors, Muscle injury, MYOD1, Perivascular Injury

## Abstract

Arteriovenous fistula (AVF) is created in end-stage renal disease patients for hemodialysis. AVF maturation failure is a common complication due to thrombosis and stenosis of the vessels involved in AVF. Chronic inflammation along with perivascular cuffing plays a critical role in AVF maturation failure. Luminal as well as periluminal factors with the involvement of transcription and epigenetic factors play a critical role in AVF maturation failure. Our previous study reported MYOD1, a factor associated with muscle regeneration and increased with muscle injury, as a significantly increased transcription factor in AVF arteries compared to control. This study aims to characterize the perivascular and vascular AVF tissues for the expression of MYOD1, mediators of inflammation, immune cell markers, and apoptosis markers with the hypothesis that muscle injury mediates the recruitment of immune cells and activation of apoptosis leading to increased apoptosis and vascular thrombosis and fibrosis of perivascular structures. Previously collected tissues from Yucatan miniswine were stained and analyzed, and the results revealed increased expression of mediators of inflammation, immune cell markers, and apoptosis markers in association with increased MYOD1 expression. The findings suggest an interaction between the expression of perivascular factors and luminal factors, but further investigations using in-vitro studies are needed to establish this relationship.

## 1. Introduction

Arteriovenous fistula (AVF) is an artificial connection made surgically to connect the artery and vein. Its main purpose is to serve as an access site for patients who need long-term hemodialysis (HD). Patients who are nearing end-stage renal disease (ESRD) are recommended to get this surgical procedure done around 6 months before HD is started [1]. Even with this recommendation, around 62.5% of patients starting on HD exclusively use a central venous catheter (CVC) to receive treatments. CVCs put a patient at a higher risk of blood infections. Combined with their medical history (obesity, type 2 diabetes, chronic kidney disease, or renal failure), sole use of CVC for dialysis puts them at a higher mortality risk. While the AVF is ideal for long-term vascular access, it also comes with post-surgical complications and a maturation rate of only 67% at 6 months [2]. Outflow vessel obstruction caused by stenosis, thrombosis, and failure to reach maturity accounts for 20–54% of primary AVF failure cases [3]. Our previous studies suggest that perivascular cuffing in the presence of inflammation contributes to AVF failure [4,5]. Fistulas that do not reach maturation cannot be used for HD. The National Kidney Foundation’s Kidney Dialysis Outcomes Quality Initiative (NKF KDOQI) defines maturation of AVF in humans by the following criteria: fistula blood flow of 600 mL/min, vein diameter of 6 mm, and depth of 2 mm below the skin [6]. Many factors are involved in the functional maturation of AVFs, however, younger patients, low BMI, male, and without vascular diseases or diabetes are likely to have a higher rate of AVF maturation [6]. Stenosis, the narrowing of an artery, is related to the inward modeling of the vessel’s neointimal layer, which can significantly reduce lumen size and blood flow. This process, often triggered by inflammation following AVF surgery, is initiated by the release of cytokines from damaged tissues and muscles at the surgical site [7]. Narrowing of vessels results in increased blood pressure, which over time causes shear stress and arterial injury that exacerbate further inflammation and remodeling in the area. Thrombosis is another major cause of AVF failure and is driven by Virchow’s Triad (endothelial injury, hypercoagulability, and stasis of blood flow). Chronic inflammation after the surgery in addition to comorbidities in ESRD patients further increases the risk of atherosclerotic plaque, thrombosis, and eventually AVF failure [4,8–10].

Inflammation after AVF creation may be due to vascular factors as well as perivascular factors such as muscle exploration/injury during AVF creation (as in the case of AVF creation involving femoral vessels in swine because of the deeper locations off femoral vessels), changed metabolomics, oxidative stress, or decreased blood supply (ischemia) after AVF creation [11,12]. These changes in muscles adjoining vessels may affect AVF maturation. After surgery, myogenic differentiation level 1 (MYOD1) rapidly increases due to muscle injury as reflected in our findings previously [4,9]. This can be as early as 6 hours, with peak levels at 24–48 hours post-surgery. Thus, it can be said that MYOD1 is an early marker in muscle injury and regeneration. The increase in MYDO1 expression is primarily observed in mononuclear cells in the injured muscle tissue, and surprisingly, these cells are usually located away from the direct site of injury. This is because MYDO1 is expressed in satellite cells, which are also known as muscle stem cells that eventually proliferate into skeletal muscle cells [13]. While MYOD1 is widely known to be a transcription factor (TF) itself, it also can recruit other TFs like c-Jun, Jdp2, Meis, and Runx1 to enhancer regions in muscle-related genes, which promotes muscle development [14]. The role of these TFs in AVF maturation has been discussed [4,9]. This study aims to characterize the AVF tissues collected from an ongoing study for the expression of MYOD1 and its association with apoptosis.

## 2. Material and Methods

### 2.1 Tissues:

AVF tissues collected from Yucatan miniswine being used for other studies in our laboratory (IACUC # R20IACUC038) [4,8,9,15] were used in this study. Femoral arteries from the control group and swine treated with TAK-242, an inhibitor of toll-like receptor-4 (TLR-4) [8]. Additionally, the tissues surrounding AVF (perivascular tissue) were also used for the expression of MYOD1.

### 2.2 Immunohistochemistry:

Arteriovenous fistula (AVF) tissues embedded in paraffin for long-term preservation were sectioned using a Leica microtome to make 5μm sections placed on a glass slide and baking at 60°C for one hour. The sections were deparaffinized using xylene and then rehydrated through a graded ethanol series (100%, 95%, 80%, 70%) before being rinsed in deionized water. Antigen retrieval was achieved using a 1% citrate buffer. The slides were washed with phosphate-buffered saline (PBS) for 5 minutes, and the tissue samples were circumscribed with a paraffin wax pen. The slides were then incubated in a 1% hydrogen peroxide (H₂O₂) solution for 15 minutes and washed twice with PBS for 5 minutes each. The blocking was performed using a blocking solution and incubated for one hour at room temperature. After removing the blocking solution, primary antibodies (Caspase-1 (ThermoFisher #14F468), Caspase-3 (Cell Signaling, 8G10 Rabbit mAb #9665), CD16 (ThermoFisher ASH 1975), IL-18 (Abcam, ab223293), neural cell adhesion molecule 1 (NCAM1) (Abcam, ab75813), MYOD1 (NovusBio NB100–56511), and interferon (IFN)-γ (AbCam ab9567)) with 1:50 dilution were applied to the tissues, followed by overnight incubation at 4°C. Post-incubation, the slides were washed twice with PBS, and the corresponding secondary antibody (Vectastain ABC kit) was added and incubated for one hour. The slides were then washed again before the ABC solution was applied, with a subsequent 30-minute incubation. An AEC solution was added for 2 minutes until a red color developed. The slides were then counterstained with hematoxylin, rinsed with water, and allowed to air dry before being mounted with Cytoseal. All stained sections were scanned at 200X using a light microscope (Leica DM6).

### 2.3 Polymerase chain reaction:

Prior to performing PCR, cDNA was synthesized from mRNA isolated from AVF tissues collected in RNA later using TRIZOL (MilliporeSigmaT9424). Quantitative real-time PCR (qRT-PCR) was conducted with the following cycling parameters: initial denaturation at 95°C for 5 minutes, followed by 40 cycles of denaturation at 95°C for 30 seconds, annealing at 55–60°C for 30 seconds, and extension at 72°C for 30 seconds. This was followed by a melting curve analysis. Reactions were performed in triplicate using SYBR Green Master Mix and a CFX96 Real-Time PCR system (BioRad Laboratories, Hercules, CA, USA). The primers used in this study were purchased from Integrated DNA Technologies (Table 1). Data was normalized to the expression of the 18S rRNA housekeeping gene and fold change in gene expression was calculated using 2^−^^CT^.

### 2.4 Statistical analysis

The data are presented as mean ± SD. To compare the fold-change in gene expression between the two groups Student’s *t*-test was used for statistical analysis. A p-value of <0.05 was considered statistically significant. Significance levels are indicated as follows: * p < 0.05, ** p < 0.01, *** p < 0.001, and **** p < 0.0001.

## 3. Results

### 3.1 Gene expression analysis revealed increased expression of factors associated with muscle regeneration, inflammation, and apoptosis:

**Figure 1:**
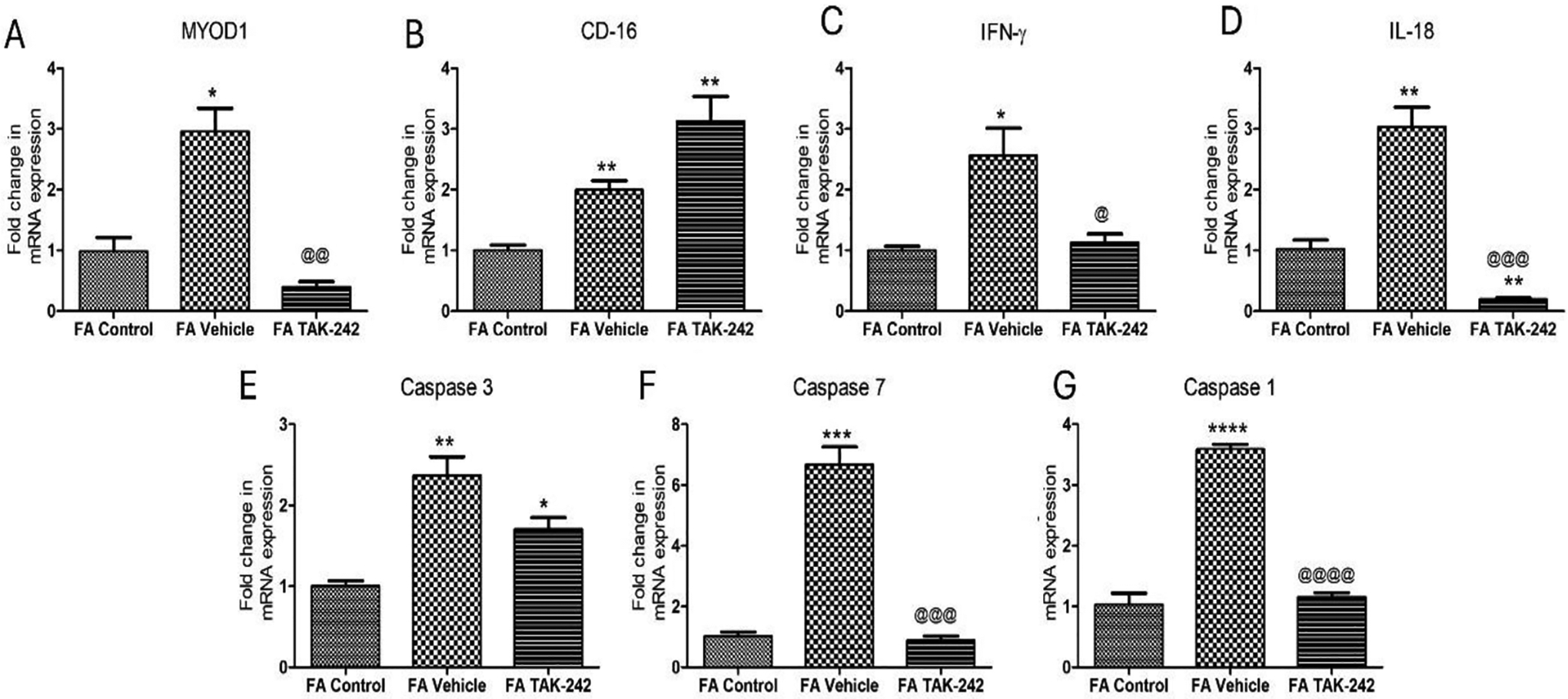
Gene expression analysis for the expression of MYOD1 (panel A), CD-16 (panel B), ING-γ (panel C), IL-18 (panel D), Caspase-1 (panel G), caspase-3 (panel E), and caspase-7 (panel F) in AVF tissues and arteries using polymerase chain reaction. The data are presented as mean ± SD. * p < 0.05, ** p < 0.01, *** p < 0.001, and **** p < 0.0001. *compared to control, ^@^compared to vehicle treated arteries.

Real-time polymerase chain reaction for the fold change in gene expression in femoral arteries (FA) without any intervention (contralateral femoral artery), FA treated with vehicle control and TLR-4 inhibitor TAK-242 revealed significantly increased expression of MYOD-1, IFN-γ, IL-18, Caspase-3, caspase-1, and caspase-7 in arteries treated with vehicle control compared to contralateral and TAK-242 treated arteries (Figure 1, panels A, C-G, respectively). The expression of CD-16 was significantly higher in vehicle-treated and TAK-242-treated arteries compared to control and in TAK-242 treated compared to vehicle-treated arteries (Figure 1 panel B). The fold change in gene expression of MYOD-1, IFN-γ, IL-18, caspase-1, and caspase-7 was significantly decreased in TAK-242-treated arteries compared to vehicle-treated arteries (Figure 1).

### 3.2 Immunostaining (IHC) revealed significantly increased expression of MYOD1, CD-16, and NCAM-1 in vehicle-treated AVF tissues:

**Figure 2:**
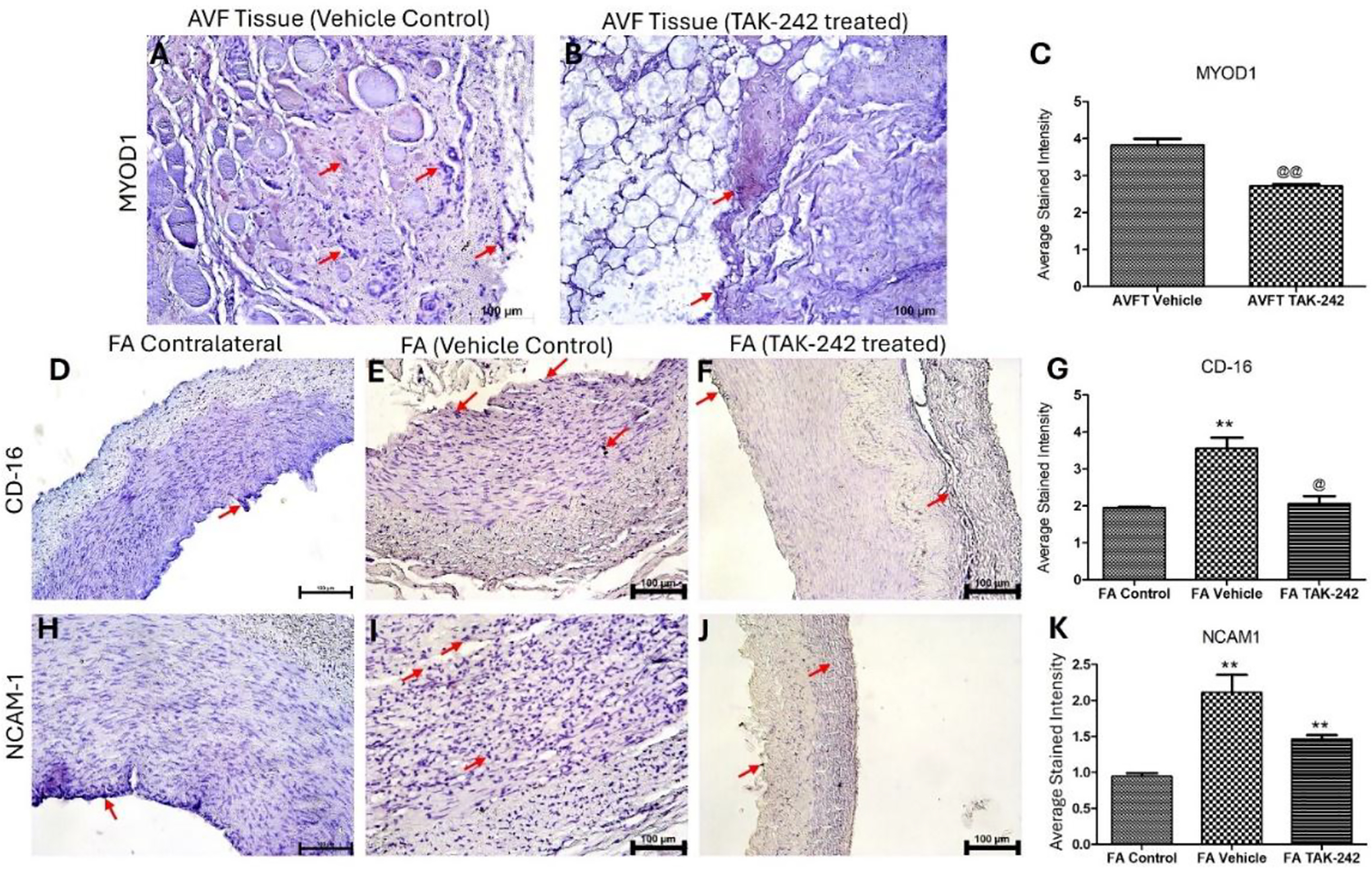
Immunohistochemistry (IHC) for MYOD1 in AVF tissues, and for CD-16 and NCAM-1 in AVF femoral arteries. IHC for MYOD1 in AVF tissues treated with vehicle control (A), TAK-242 (B), CD-16 in femoral arteries (D-F), and NCAM-1 (H-J), and average stained intensity (C, G, and K). The data are presented as mean ± SD. * p < 0.05, ** p < 0.01, *** p < 0.001, and **** p < 0.0001. *compared to control, ^@^compared to vehicle treated arteries. The red arrow points to positive staining.

IHC of tissues around AVF showed increased MYOD1 expression in tissues surrounding AVF (perivascular) treated with vehicle control compared to TAK-242 treated AVF injected during AVF creation (Figure 2 panels A-C). In the arteries treated with vehicle control, the expression of CD-16 and NCAM-1 was significantly increased compared to control arteries (Figure 2 panels D-K). The expression of CD-16 in TAK-242 treated arteries was significantly decreased compared to vehicle-treated arteries (Figure 2 panel G) while the expression of NCAM-1 was significantly increased compared to control arteries (Figure 2 panel K).

### 3.3 Immunostaining (IHC) revealed significantly increased expression of IFN-γ and IL-18 in vehicle-treated AVF tissues:

**Figure 3:**
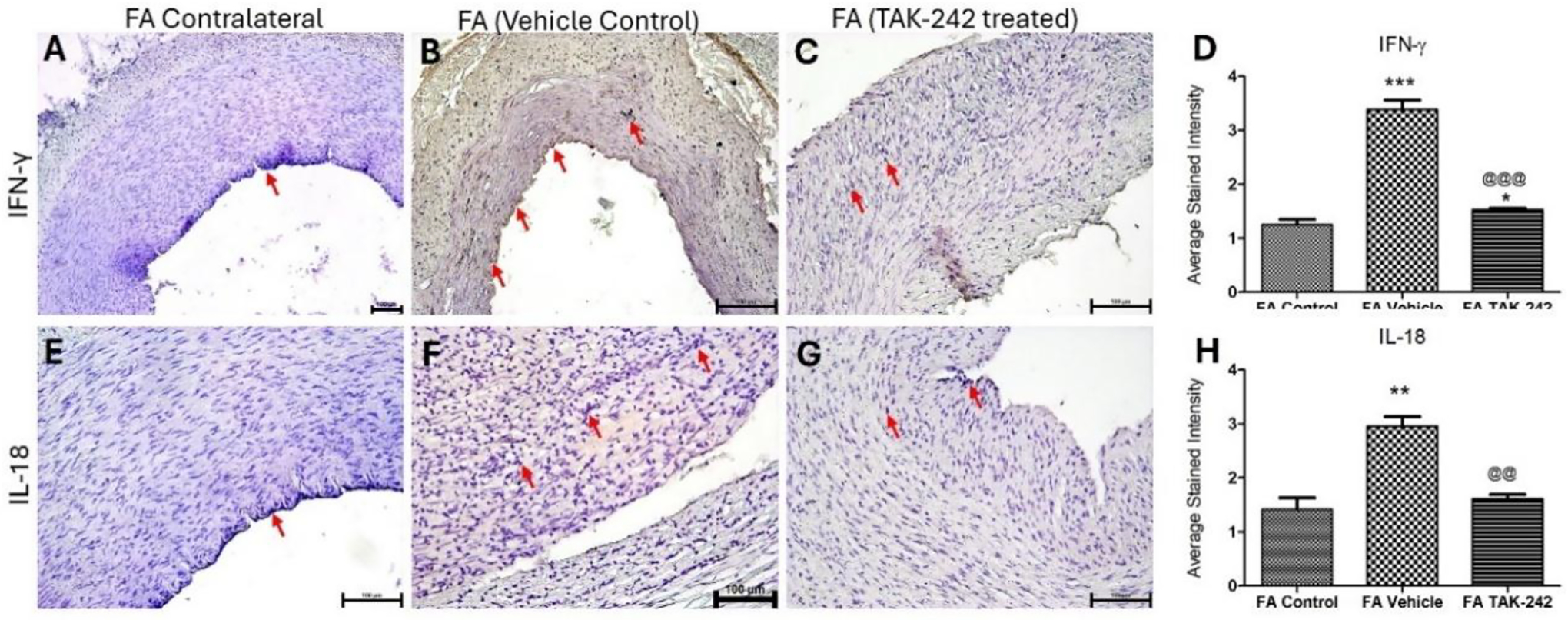
Immunohistochemistry (IHC) for IFN-γ and IL-18 in AVF femoral arteries. IHC for IFN-γ (A-C) and NCAM-1 (E-G) in femoral arteries and average stained intensity (D and H). The data are presented as mean ± SD. * p < 0.05, ** p < 0.01, *** p < 0.001, and **** p < 0.0001. *compared to control, ^@^compared to vehicle treated arteries. The red arrow points to positive staining.

IHC of the arteries treated with vehicle control revealed significantly increased expression of IFN-γ and IL-18 compared to control arteries (Figure 3 panels A-H). The expression of IFN-γ (Figure 2 panel D) and IL-18 (Figure 3 panel H) in TAK-242-treated arteries was significantly decreased compared to vehicle-treated arteries.

### 3.4 Immunostaining (IHC) revealed significantly increased expression of caspase-1 and caspase-3 in vehicle-treated AVF tissues:

IHC of the arteries treated with vehicle control revealed significantly increased expression of caspase-1 and caspase-3 compared to control arteries (Figure 3 panels A-H). The expression of caspase-1 (Figure 2 panel D) and caspase-3 (Figure 3 panel H) in TAK-242 treated arteries was significantly decreased compared to vehicle-treated arteries.

## 4. Discussion

The damaged tissue after a muscle injury releases signals triggering an immune response resulting in the recruitment of immune cells including neutrophils, macrophages, and lymphocytes to the injury site. The immune cells are involved in clearing the debris via phagocytose and this starts the repair followed by regeneration of the tissue. During this repair process, many of the recruited immune cells undergo apoptosis which is crucial for proper muscle healing and preventing excessive inflammation. The apoptotic immune cells signal to other cells like satellite cells to initiate muscle regeneration, however, disruption or alteration of the apoptotic process can lead to excessive inflammation and impaired muscle repair [16–19]. Further, increased smooth muscle cell apoptosis during vessel remodeling can contribute to the changes in the structure and function of the vessel associated with atherosclerosis because increased apoptosis facilitates the removal of old or damaged cells and the proliferation of new cells involved in vessel remodeling [20]. Previously we reported that perivascular cuffing in the presence of inflammation plays a critical role in early thrombosis in AVF [4] and MYOD1 is a differentially expressed gene after AVF creation [9]. The results of this study revealed an increased expression of MYOD1 in tissues around AVF treated with vehicle compared to TAK-242. This suggests that attenuating inflammation promotes remodeling.

MYDO1 is responsible for being the “genome organizer” that specifies the three-dimensional architecture unique to muscle cell development. This highlights the significant role of MYOD1 in establishing and maintaining the muscle cell’s identity throughout the remodeling process [21]. MYDO1 expression occurs alongside the inflammatory response involving the recruitment of pro-inflammatory macrophages (M1 macrophages) to the site of injury, and satellite cells begin to activate and proliferate. Then, when anti-inflammatory macrophages (M2 macrophages) are activated to promote healing and reduce inflammation, muscle differentiation begins. Any dysregulation in this process leads to attenuation or reduction of muscle regeneration, as well as a reduction in the growth of muscle fibers. This suggests that MYOD1 must work in synchrony with the inflammatory response to promote healing [22]. The inflammatory response is mediated by recruited immune cells. The results of this study showed increased expression of CD-16 and NCAM-1, the markers of neutrophils, suggesting recruitment of neutrophils in the arteries involved in AVF. We previously reported the recruitment of monocytes, macrophages, dendritic cells, and lymphocytes in the vessels after AVF creation [15]. The recruitment of these immune cells is mediated by injury to the vessels as well as muscle injury as evidenced by increased MYOD1. This suggests that injury to the perivascular tissues may affect AVF maturation. Persistent inflammation recruiting more immune cells enables a positive feedback loop of inflammatory response leading to remodeling [5]. Thus, to increase the chances of AVF maturation, it is important to regulate inflammation by having a balance of both pro and anti-inflammatory pathways.

Increased inflammation after AVF creation is evidenced by our previous findings of increased IL-6 and TNF-α and immune cell recruitment in control groups which are attenuated by inhibition of TLR-4 using TAK-242 [8,15]. This study also showed an increased expression of IFN-γ and IL-18 in arteries treated with vehicle control compared to TAK-242-treated arteries. TAK-242, a TLR-4 inhibitor, was found to attenuate inflammation, which can contribute to the prevention of early thrombosis in AVF patients [8]. Attenuating inflammation not only promotes AVF maturation but can also promote muscle repair. Though a certain level of inflammation is necessary for muscle regeneration, excessive or prolonged inflammation can hinder muscle repair and promote fibrosis. Therefore, reducing inflammation to a controlled level can promote muscle regeneration [22,23] and a decreased level and expression of MYOD1 in TAK-242-treated swine support the notion that inhibiting inflammation supports muscle repair. Overall, by reducing inflammation, TAK-242 may create a more favorable environment for AVF maturation and muscle repair.

Apoptosis, crucial for vascular remodeling and muscle repair, is a noninflammatory process under normal conditions however, can become inflammatory under certain conditions including the release of damage-associated molecular patterns after injury and increased expression of TLR-4 due to increased secretion of cytokines [24–26]. Increased TLR-4 expression is associated with AVF creation and stenosis and thrombosis of AVF vessels [8]. Further, TLR-4 can promote caspase activity and TLR-4 signaling is associated with the activation of caspase-4, caspase-11, caspase-1, caspase-3, caspase-7, and caspase-8 involving IL-18, IL-1β, and PPAR activation contributing to caspase-mediated apoptosis, mitochondrial dysfunction, fibrosis, and inflammation [27–30]. In this study, we found increased expression of caspase-1, caspase-3, and caspase-7 in AVF arteries treated with vehicle control compared to control and TAK-242-treated arteries. It was also evident that TAK-242 inhibiting TLR-4 attenuated the expression of caspase-1, −3, and −7. These findings suggest that inhibiting inflammation may also regulate apoptosis by regulating caspase expression.

The association of increased MYOD1 with increased expression of CD-16, IFN-γ, IL-18, and caspases in this study suggests that injury to the muscle and fascia adjoining to the vessels involved in AVF creation may affect the expression of various factors mediating vessel remodeling. In other words, luminal and perivascular factors may play crucial roles in the maturation of AVFs. Luminal factors are elements within a vessel, such as the pressure gradient, vessel wall shear stress, and characteristics of blood flow. Perivascular factors refer to elements surrounding a vessel, like perivascular cuffing, surrounding tissue factors, and the extracellular matrix (ECM) [4,8,9,31]. Neointimal hyperplasia (NIH), is a pathological condition in which vascular smooth muscle cells (VSMCs) proliferate within a vessel and thicken it. It does so by influencing the migration of fibroblasts and smooth muscle cells to the intimal layer. This result is a combination of luminal factors like shear stress on the vessel wall as well as perivascular factors like local inflammation [32], both playing a critical role in remodeling.

## 5. Conclusion

The results of this study suggest an association between the expression of luminal and perivascular factors associating apoptosis with the presence of inflammation. The findings suggest that apoptosis may play a critical role in the AVF maturation process with an effect on perivascular structures. However, it is not clear whether the increase in caspases is due to injury to perivascular structures or due to injury to the vessels during AVF creation. In-vitro studies involving cells treated with cytokines and growth factors with co-culture studies of myocytes vascular smooth muscle cells and endothelial cells are needed to corroborate these findings.

## Figures and Tables

**Figure 4:**
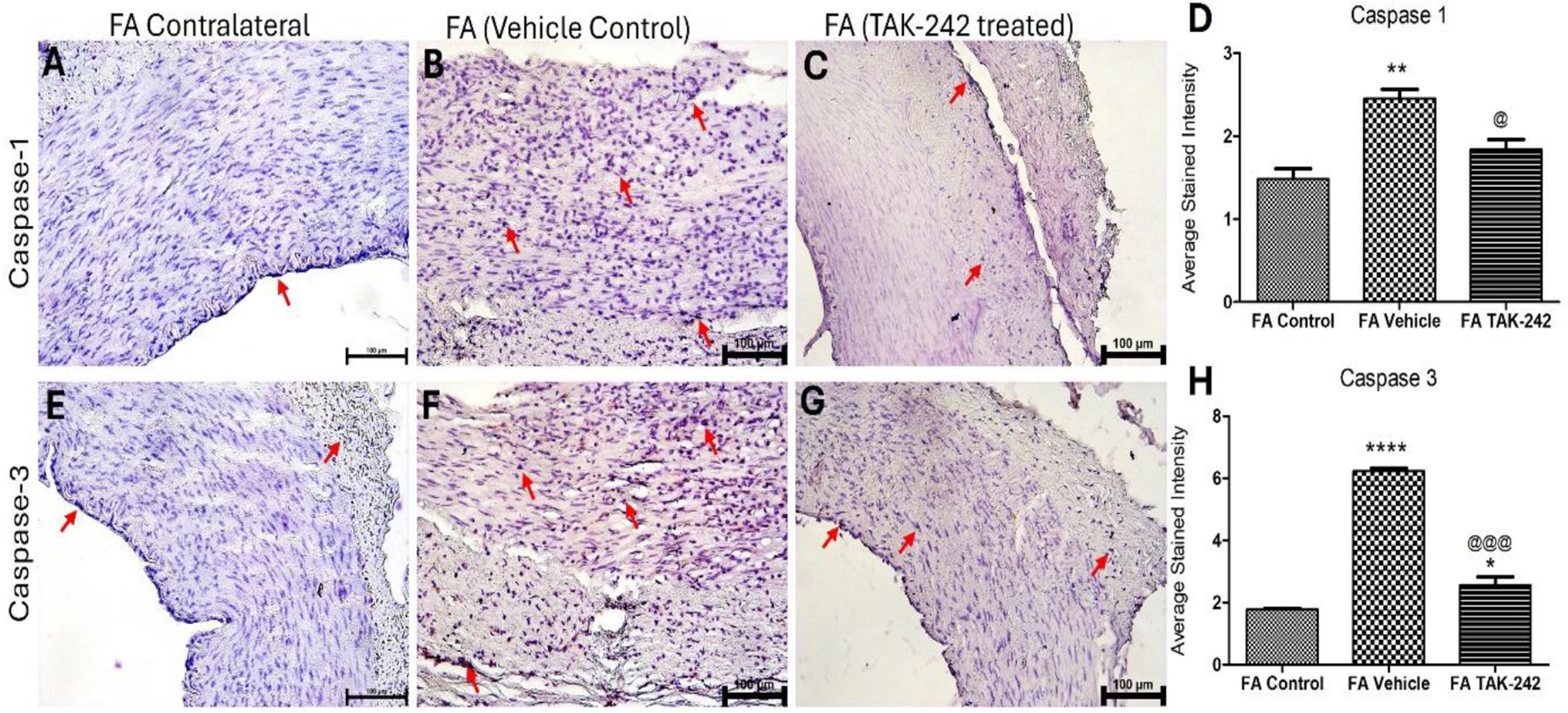
Immunohistochemistry (IHC) for caspase-1 and caspase-3 in AVF femoral arteries. IHC for caspase-1 (A-C) and caspase-3 (E-G) in femoral arteries and average stained intensity (D and H). The data are presented as mean ± SD. * p < 0.05, ** p < 0.01, *** p < 0.001, and **** p < 0.0001. *compared to control, ^@^compared to vehicle treated arteries. The red arrow points to positive staining.

**Table 1:** Forward and reverse sequence of the primer nucleotides used in this study.

Gene Name	Forward Primer	Reverse Primer
MYOD1	5’ - TGC TAC GAC GGC ACC TAT TA-3’	5’- AGA TGC TCT CCA CGA TGC T-3’
IFNG	5’ - GGT AGC TCT GGG AAA CTG AAT G-3’	5’- CTG ACT TCT CTT CCG CTT TCT T –3’
IL18	5’-GTA GCT GAA AAC GAT GAA GAC CTG-3’	5’- GGC ATA TCC TCA AAC ACG GC –3
sCASP3	5’–ACG GAC AGT GGG ACT GAA GAT G-3’	5’-ACT GGA TGA ACC AGG ATC CGT C-3’
CD16	5’- CTT TCT ACC TTG GCA CCA AAT C-3’	5’ - CTC CAT GTG ACT TTG CCA TTC –3’
CASP 1	5’- GGG TTA CAG TGT GGA TGT TAG AG-3’	5’-CAT GAG ACA TGA GCA CCA GAA-3
18S	5’-CCCACGGAATCGAGAAAGAG-3’	5’-TTGACGGAAGGGCACCA-3’

## Data Availability

All the analyzed data have been included in the manuscript. The raw data will be provided by the corresponding author on request.

## References

[1] DeVita MV, Khine SK, Shivarov H. Novel Approaches to Arteriovenous Access Creation, Maturation, Suitability, and Durability for Dialysis. Kidney Int Rep 5 (2020): 769–78. 32518859 10.1016/j.ekir.2020.02.1024 PMC7270716

[2] Huber TS, Berceli SA, Scali ST, Arteriovenous Fistula Maturation, Functional Patency, and Intervention Rates. JAMA Surg 156 (2021): 1111–8. 34550312 10.1001/jamasurg.2021.4527 PMC8459303

[3] Siddiqui MA, Ashraff S, Carline T. Maturation of arteriovenous fistula: Analysis of key factors. Kidney Res Clin Pract 36 (2017): 318–28. 29285424 10.23876/j.krcp.2017.36.4.318 PMC5743041

[4] Rai V, Agrawal DK. Transcriptomic Analysis Identifies Differentially Expressed Genes Associated with Vascular Cuffing and Chronic Inflammation Mediating Early Thrombosis in Arteriovenous Fistula. Biomedicines 10 (2022). 10.3390/biomedicines10020433 PMC8962355 35203642

[5] Xia F, Rai V, Agrawal DK. Vascular and Perivascular Role in the Regulation of Angiogenesis: Impact on Arteriovenous Fistula Maturation. Archives of Internal Medicine Research 7 (2024): 284–96. 39698202 10.26502/aimr.0185 PMC11654682

[6] Lok CE, Huber TS, Lee T, KDOQI Clinical Practice Guideline for Vascular Access: 2019 Update. Am J Kidney Dis 75 (2020): S1–S164. 10.1053/j.ajkd.2019.12.001 32778223

[7] Laboyrie SL, de Vries MR, Bijkerk R, Building a Scaffold for Arteriovenous Fistula Maturation: Unravelling the Role of the Extracellular Matrix. Int J Mol Sci 24 (2023). 10.3390/ijms241310825 PMC10341877 37446003

[8] Rai V, Radwan MM, Nooti S, TLR-4 Inhibition Attenuates Inflammation, Thrombosis, and Stenosis in Arteriovenous Fistula in Yucatan Miniswine. Cardiol Cardiovasc Med 6 (2022): 432–50. 36147190 10.26502/fccm.92920280 PMC9491704

[9] Rai V, Agrawal DK. Transcriptional and Epigenetic Factors Associated with Early Thrombosis of Femoral Artery Involved in Arteriovenous Fistula. Proteomes 10 (2022). 10.3390/proteomes10020014 PMC9149803 35645372

[10] MacRae JM, Dipchand C, Oliver M, Arteriovenous Access Failure, Stenosis, and Thrombosis. Can J Kidney Health Dis 3 (2016): 2054358116669126. 10.1177/2054358116669126 PMC5332078 28270918

[11] Sangani V, Pokal M, Noel E, A Case of Ischemic Monomelic Neuropathy After Arteriovenous Fistula Placement. J Community Hosp Intern Med Perspect 13 (2023): 113–7. 37877056 10.55729/2000-9666.1200 PMC10593159

[12] Khattri RB, Kim K, Anderson EM, Metabolomic profiling reveals muscle metabolic changes following iliac arteriovenous fistula creation in mice. Am J Physiol Renal Physiol 323 (2022): F577–F89. 36007889 10.1152/ajprenal.00156.2022 PMC9602894

[13] Grounds MD, Garrett KL, Lai MC, Identification of skeletal muscle precursor cells in vivo by use of MyoD1 and myogenin probes. Cell Tissue Res 267 1992 (1): 99–104. 1310442 10.1007/BF00318695

[14] Blum R, Dynlacht BD. The role of MyoD1 and histone modifications in the activation of muscle enhancers. Epigenetics 8 (2013): 778–84. 23880568 10.4161/epi.25441 PMC3883780

[15] Samra G, Rai V, Agrawal DK. Heterogeneous Population of Immune cells Associated with Early Thrombosis in Arteriovenous Fistula. J Surg Res (Houst) 5 (2022): 423–34. 35937643 10.26502/jsr.10020237 PMC9354142

[16] Sciorati C, Rigamonti E, Manfredi AA, Cell death, clearance and immunity in the skeletal muscle. Cell Death Differ 23 (2016): 927–37. 26868912 10.1038/cdd.2015.171 PMC4987728

[17] Ziemkiewicz N, Hilliard G, Pullen NA, The Role of Innate and Adaptive Immune Cells in Skeletal Muscle Regeneration. Int J Mol Sci 22 (2021). 10.3390/ijms22063265 PMC8005179 33806895

[18] Wu J, Ren B, Wang D, Regulatory T cells in skeletal muscle repair and regeneration: recent insights. Cell Death Dis 13 (2022): 680. 35931697 10.1038/s41419-022-05142-8 PMC9356005

[19] Koike H, Manabe I, Oishi Y. Mechanisms of cooperative cell-cell interactions in skeletal muscle regeneration. Inflamm Regen 42 (2022): 48. 36380396 10.1186/s41232-022-00234-6 PMC9667595

[20] Yu H, Clarke MC, Figg N, Smooth muscle cell apoptosis promotes vessel remodeling and repair via activation of cell migration, proliferation, and collagen synthesis. Arterioscler Thromb Vasc Biol 31 (2011): 2402–9. 21885847 10.1161/ATVBAHA.111.235622

[21] Wang R, Chen F, Chen Q, MyoD is a 3D genome structure organizer for muscle cell identity. Nat Commun 13 (2022): 205. 35017543 10.1038/s41467-021-27865-6 PMC8752600

[22] Howard EE, Pasiakos SM, Blesso CN, Divergent Roles of Inflammation in Skeletal Muscle Recovery From Injury. Front Physiol 11 (2020): 87. 32116792 10.3389/fphys.2020.00087 PMC7031348

[23] Peake JM, Neubauer O, Della Gatta PA, Muscle damage and inflammation during recovery from exercise. J Appl Physiol 122 (2017): 559–70. 28035017 10.1152/japplphysiol.00971.2016

[24] Bock FJ, Riley JS. When cell death goes wrong: inflammatory outcomes of failed apoptosis and mitotic cell death. Cell Death Differ 30 (2023): 293–303. 36376381 10.1038/s41418-022-01082-0 PMC9661468

[25] Vringer E, Tait SWG. Mitochondria and cell death-associated inflammation. Cell Death Differ 30 (2023): 304–12. 36447047 10.1038/s41418-022-01094-w PMC9950460

[26] Li C, Che LH, Ji TF, Effects of the TLR4 signaling pathway on apoptosis of neuronal cells in diabetes mellitus complicated with cerebral infarction in a rat model. Sci Rep 7 (2017): 43834. 28272417 10.1038/srep43834 PMC5341048

[27] Eltom S, Belvisi MG, Yew-Booth L, TLR4 activation induces IL-1beta release via an IPAF dependent but caspase 1/11/8 independent pathway in the lung. Respir Res 15 (2014): 87. 25085021 10.1186/s12931-014-0087-0 PMC4347603

[28] Hsu HY, Lin TY, Hu CH, Fucoidan upregulates TLR4/CHOP-mediated caspase-3 and PARP activation to enhance cisplatin-induced cytotoxicity in human lung cancer cells. Cancer Lett 432 (2018): 112–20. 29746926 10.1016/j.canlet.2018.05.006

[29] Adanitsch F, Shi J, Shao F, Synthetic glycan-based TLR4 agonists targeting caspase-4/11 for the development of adjuvants and immunotherapeutics. Chem Sci 9 (2018): 3957–63. 29780528 10.1039/c7sc05323a PMC5941199

[30] Katare PB, Nizami HL, Paramesha B, Activation of toll like receptor 4 (TLR4) promotes cardiomyocyte apoptosis through SIRT2 dependent p53 deacetylation. Sci Rep 10 (2020): 19232. 33159115 10.1038/s41598-020-75301-4 PMC7648754

[31] DeMarco N, Rai V, Wilson DR, Oncostatin M, Serpins, and Oxidative Stress in Extracellular Matrix Remodeling and Arteriovenous Fistula Maturation. Cardiol Cardiovasc Med 7 (2023): 129–40. 37484520 10.26502/fccm.92920318 PMC10361734

[32] Barcena AJR, Perez JVD, Liu O, Localized Perivascular Therapeutic Approaches to Inhibit Venous Neointimal Hyperplasia in Arteriovenous Fistula Access for Hemodialysis Use. Biomolecules 12 (2022). 10.3390/biom12101367 PMC9599524 36291576

